# Ammonia production by human faecal bacteria, and the enumeration, isolation and characterization of bacteria capable of growth on peptides and amino acids

**DOI:** 10.1186/1471-2180-13-6

**Published:** 2013-01-11

**Authors:** Anthony J Richardson, Nest McKain, R John Wallace

**Affiliations:** 1 Rowett Institute of Nutrition and Health, University of Aberdeen, Bucksburn, Aberdeen, AB21 9SB, UK

**Keywords:** *Clostridium perfringens*, Colonic bacteria, Deamination, Peptide metabolism

## Abstract

**Background:**

The products of protein breakdown in the human colon are considered to be detrimental to gut health. Amino acid catabolism leads to the formation of sulfides, phenolic compounds and amines, which are inflammatory and/or precursors to the formation of carcinogens, including N-nitroso compounds. The aim of this study was to investigate the kinetics of protein breakdown and the bacterial species involved.

**Results:**

Casein, pancreatic casein hydrolysate (mainly short-chain peptides) or amino acids were incubated *in vitro* with suspensions of faecal bacteria from 3 omnivorous and 3 vegetarian human donors. Results from the two donor groups were similar. Ammonia production was highest from peptides, followed by casein and amino acids, which were similar. The amino acids metabolized most extensively were Asp, Ser, Lys and Glu. Monensin inhibited the rate of ammonia production from amino acids by 60% (*P* = 0.001), indicating the involvement of Gram-positive bacteria. Enrichment cultures were carried out to investigate if, by analogy with the rumen, there was a significant population of asaccharolytic, obligately amino acid-fermenting bacteria (‘hyper-ammonia-producing’ bacteria; HAP) in the colon. Numbers of bacteria capable of growth on peptides or amino acids alone averaged 3.5% of the total viable count, somewhat higher than the rumen. None of these were HAP, however. The species enriched included *Clostridium* spp., one of which was *C*. *perfringens*, *Enterococcus*, *Shigella* and *Escherichia coli*.

**Conclusions:**

Protein fermentation by human faecal bacteria in the absence of sugars not only leads to the formation of hazardous metabolic products, but also to the possible proliferation of harmful bacteria. The kinetics of protein metabolism were similar to the rumen, but HAP bacteria were not found.

## Background

Protein is an abundant substrate for bacterial growth in the human intestine, possibly more so than carbohydrate in the distal colon [1]. Some of the protein may be of dietary origin, but large intestinal fermentation probably depends more on endogenous sources, including mucus and host proteins and bacterial protein resulting from bacterial cell turnover. The metabolism of protein and its peptide and amino acid hydrolysis products by colonic bacteria can lead to the formation of several by-products that may be hazardous to health [2]. N-nitroso compounds are formed from amines and amides, which in turn arise from the metabolism of amino acids; they are heavily implicated in the etiology of colorectal cancer [3]. Hydrogen sulfide is a product of the breakdown of cysteine and methionine; sulfides induce hyperproliferation of crypt cells [4], and predispose to colonic carcinomas [5] and ulcerative colitis [6]. Other potentially toxic products of protein breakdown in the large intestine include phenols, ammonia and indoles [7]. Thus, understanding the processes and bacteria that carry out proteolysis and its subsequent reactions is highly relevant to human gut health.

Proteolytic species from the human colon have been well characterized [1,8,9], and some aspects of the metabolism of peptides are known [1,10]. Bacterial species able to grow on individual amino acids as N and energy source are fairly well understood [11]. They include many of the ‘putrefactive’ *Clostridium*, *Peptostreptococcus* and *Fusobacterium* species [11,12]. Some evidence that gut bacteria can also use Stickland reactions, which involves the coupled oxidation and reduction of pairs of amino acids to organic acids [13], was obtained by Smith and Macfarlane [1]. However, bacteria able to grow on a mixture of protein breakdown products, although known to be numerous [11], have not been characterized. It is possible that the species that derive energy from protein in the colon are among the most numerous species which, when carbohydrate has been exhausted, switch to amino acids as a substrate for generating metabolic energy. It is also possible, however, that a less numerous, high-activity functional group of deaminative bacteria exists, analogous to the ‘hyper-ammonia-producing’ (HAP) bacteria of the rumen [14-18]. These asaccharolytic bacteria generate NH_3_ at a rate far greater than the most numerous ruminal species, such that, although their population size is small, they may make a significant contribution to overall NH_3_ production in the rumen of cattle and sheep. Attention has been paid to these bacteria because of their impact on N retention in the animal. If they were to exist in the human colon, they might have a similar significance, except to human health rather than nutrition. They might also be subject to dietary manipulation, as in the rumen [18,19]. The aim of the present work was therefore to investigate the properties of NH_3_ production from protein in the colon, and to use methods that revealed the ruminal HAP population to determine if HAP populations also exist in the human colonic microbiota.

## Results

### Ammonia production in faecal suspensions *in vitro*

The rate of NH_3_ production by mixed faecal bacteria depended on the donor and the substrate. Six samples were investigated for their activity with Trypticase, a pancreatic casein hydrolysate containing predominantly peptides, and an amino acid mixture formulated to contain the same amino acid composition (Table 1). There were significant differences (*P* < 0.001) between production rates on Trypticase and amino acids, and the production rate was decreased by monensin (*P* < 0.001) but there was no interaction (*P* = 0.866). Activities were similar in the 3 samples from omnivores and in one sample from a vegetarian, while one vegetarian sample had about half the average activity and the other double the average. The type of subject diet did not affect production rate (*P* = 0.678). In a different set of samples from donors O1, O2 and V1, the rate of NH_3_ production from casein was 19% lower than from Trypticase (*P* = 0.04) and not different from amino acids (*P* >0.05) (results not shown). Monensin had a greater effect on NH_3_ production from amino acids (60% inhibition) compared to peptides (Trypticase; 39% inhibition) (Table 1; *P* = 0.003).

**Table 1:** **Ammonia production from peptides (Trypticase) and amino acids by mixed human faecal bacteria *in vitro *with and without added 5 μM monensin**

**Substrate**	**Rate of ammonia production**							
	**(μmol (mg protein)**^**-1**^ **h**^**-1**^**)**							
**Donor**	**O1**	**O2**	**O3**	**V1**	**V2**	**V3**	**Mean**	**SE**
Trypticase	1.44	1.39	1.62	0.65	3.03	1.71	1.64	0.39
Amino acids	1.00	0.94	1.13	0.40	2.30	1.04	1.14	0.31
Trypticase + monensin	0.88	0.80	1.01	0.50	2.04	0.80	1.00	0.27
Amino acids + monensin	0.50	0.30	0.43	0.28	0.96	0.31	0.46	0.13
*P* values								
Trypticase *vs* amino acids			<0.001					
Monensin			<0.001					
Trypticase *vs* amino acids × monensin			0.866					
O or V, Trypticase *vs* amino acids			0.648					
O or V, monensin,			0.631					

Amino acid analysis revealed that total amino acid breakdown was slightly greater with peptides than amino acids, but the effect was not significant (Table 2). No amino acid was degraded completely during the course of the incubations. The order of rates of disappearance of different amino acids was also similar for both substrates, with Glu, Asp, Ser and Lys being most rapidly degraded and the aromatic amino acids, Phe and Tyr, being most resistant to degradation. Tryptophan was not present in the analysis because it was destroyed during the acid hydrolysis method used to hydrolyse samples. Monensin had a major inhibitory effect on the breakdown of amino acids in both substrates, with an inhibition of 61% with amino acids and 48% with Trypticase (Table 2). The effects were different with different amino acids and according to the substrate. The breakdown of free Glu and Ala was completely inhibited, resulting in slight net synthesis, and Pro metabolism decreased by 86%. In contrast, breakdown of Asp in the amino acids mixture was unaffected by monensin, and Arg breakdown was inhibited only by 15% For the most part, monensin inhibited amino acid dissimilation to the same extent, whether present in peptides or amino acids. Again, Glu was an exception, its metabolism being inhibited less when present in peptide form.

**Table 2:** **Amino acid utilization from peptides (Trypticase) and amino acids by mixed human faecal bacteria *in vitro *with and without added 5 μM monensin**

	**Amino acids**		**Amino acids + monensin**		**Trypticase**		**Trypticase + monensin**			***P *value**	
	**Mean**^**a**^	**SE**	**Mean**	**SE**	**Mean**	**SE**	**Mean**	**SE**	**Trypticase *vs *amino acids**	**Effect of monensin, amino acids**	**Effect of monensin, trypticase**
**ASP**	0.673	0.171	0.650	0.170	0.754	0.159	0.570	0.160	NS	NS	0.050
**GLU**	1.460	0.367	−0.155	0.153	1.356	0.363	0.532	0.276	NS	0.005	0.006
**SER**	0.804	0.103	0.539	0.148	0.735	0.106	0.535	0.130	NS	NS	NS
**GLY**	0.414	0.086	0.056	0.044	0.386	0.052	0.092	0.039	NS	0.005	0.001
**HIS**	0.178	0.030	0.055	0.023	0.200	0.029	0.077	0.029	NS	0.006	0.018
**ARG**	0.255	0.034	0.217	0.042	0.347	0.035	0.339	0.070	NS	NS	NS
**THR**	0.361	0.083	0.156	0.047	0.626	0.063	0.343	0.080	0.005	0.023	0.007
**ALA**	0.139	0.053	−0.027	0.041	0.207	0.042	0.032	0.050	NS	0.034	0.000
**PRO**	0.468	0.157	0.067	0.100	0.685	0.171	0.055	0.094	NS	0.013	0.012
**TYR**	0.078	0.031	0.024	0.019	0.062	0.013	0.031	0.014	NS	0.015	0.009
**VAL**	0.132	0.062	0.026	0.051	0.153	0.037	0.070	0.042	NS	NS	NS
**ILE**	0.140	0.054	0.040	0.040	0.178	0.038	0.088	0.023	NS	0.022	NS
**LEU**	0.278	0.097	0.151	0.098	0.343	0.082	0.250	0.097	NS	0.025	NS
**PHE**	0.094	0.031	0.042	0.024	0.149	0.031	0.082	0.015	NS	0.014	NS
**LYS**	0.542	0.130	0.396	0.146	0.764	0.166	0.498	0.164	0.043	0.014	NS
**Total**	6.017	1.214	2.237	0.907	6.946	0.976	3.596	0.658	NS	0.011	0.005

^a^μmol amino acid metabolised h^-1^ ml^-1^, n = 6.

NS, *P* > 0.05.

### Assessment of population size of bacteria capable of growth on peptides and amino acids

**Figure 1:**
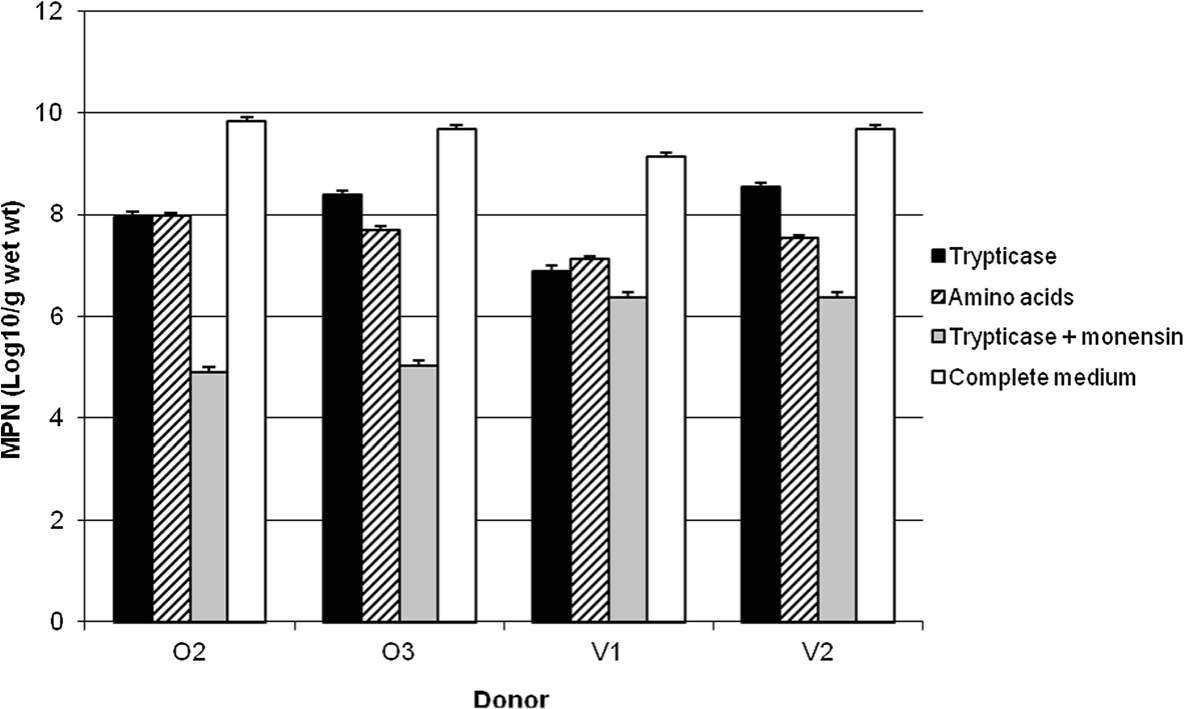
**Most-probable-numbers (MPN) counts of Trypticase and amino acid-utilising bacteria in faeces from human omnivorous (O2 and O3) and vegetarian (V1 and V2) donors.** Results are from 7-d counts. Error bars represent 95% confidence levels.

When dilutions of faecal bacteria were inoculated into liquid media in anaerobic culture tubes, both the number of tubes showing growth and the cell density achieved increased with the time of incubation. Numbers on complete medium generally reached a plateau at 2 d, while growth on the other, sugars-free media usually reached a maximum at 4 d, although because a few increases were observed between 4 and 7 d, 7-d values were used to calculate bacterial numbers (Figure 1). Total numbers approached 10^10^ (g wet wt)^-1^, while numbers capable of growing on sugar-free media after 7 d were about 10^8^ (g wet wt)^-1^. Actual counts on Trypticase medium varied from 0.8 × 10^7^ to 3.5 × 10^8^ (g wet wt)^-1^. Monensin decreased numbers in Trypticase medium by an average of 92% to 10^5^-10^6^ (g wet wt)^-1^. Amino acid utilizers were on average only slightly (26%) fewer in number than Trypticase utilizers.

### Bacterial isolates

A total of 53 isolates was isolated from the highest dilutions of faecal bacteria from two ominivores and one vegetarian. Twenty-eight survived repeated sub-culture, of which 24 gave full length or near full length 16S rRNA gene sequences (Table 3). The remaining four were identified from partial sequences. None of the isolates was asaccharolytic, growth being increased significantly in all cases by the addition of glucose to the medium. The bacteria enriched from the faecal samples appeared to be different depending on whether the substrate was peptides or amino acids. *Shigella* spp. and *E*. *coli* were more numerous in the amino acids-containing cultures. Other pathogens that were enriched included *Enterococcus faecalis*, *Staphylococcus* sp. and *Eggerthella lenta*.

**Table 3:** Identity of bacteria isolated from peptides or amino acids enrichments

**Isolate**	**Vol/ diln**	**Identification**	**% Sim**	**Phylum**	**Class**	**Order**	**Accession no.**
**Peptides**							
1	O1/5	*Clostridium perfringens*	99	Firmicutes	Clostridia	Clostridiales	GU968162
3	O1/5	*Clostridium orbiscindens*	99	Firmicutes	Clostridia	Clostridiales	GU968163
5	O1/5	*Shigella sonnei*	98	Proteobacteria	Gammaproteobacteria	Enterobacteriales	GU968164
6	O1/6	*Enterococcus faecium*	99	Firmicutes	Bacilli	Lactobacillales	GU968165
8	O1/6	*Bacteroides ovatus*	99	Bacteroidetes	Bacteroidia	Bacteroidales	GU968166
12	O2/5	*C. orbiscindens*	97	Firmicutes	Clostridia	Clostridiales	893 bp
13	O2/5	*Clostridium innocuum*	98	Firmicutes	Clostridia	Clostridiales	GU968167
14	O2/5	*B. ovatus*	93	Bacteroidetes	Bacteroidia	Bacteroidales	GU968168
15	O2/5	*Blautia hydrogenotrophica*	95	Firmicutes	Clostridia	Clostridiales	GU968169
16	O2/6	*C. orbiscindens*	95	Firmicutes	Clostridia	Clostridiales	877 bp
17	O2/6	*C. orbiscindens*	99	Firmicutes	Clostridia	Clostridiales	GU968170
21	V1/5	*Bacteroides fragilis*	99	Bacteroidetes	Bacteroidia	Bacteroidales	GU968171
22	V1/5	*Escherichia coli*	99	Proteobacteria	Gammaproteobacteria	Enterobacteriales	GU968172
23	V1/5	*B. fragilis*	98	Bacteroidetes	Bacteroidia	Bacteroidales	GU968173
25	V1/6	*B. fragilis*	99	Bacteroidetes	Bacteroidia	Bacteroidales	GU968174
27	V1/6	*E. faecium*	99	Firmicutes	Bacilli	Lactobacillales	GU968175
**Amino acids**							
29	O1/6	*Shigella* sp.	100	Proteobacteria	Gammaproteobacteria	Enterobacteriales	GU968176
31	O1/6	*Staphylococcus* sp.	99	Firmicutes	Bacilli	Bacillales	GU968177
33	O1/7	*Shigella flexneri*	98	Proteobacteria	Gammaproteobacteria	Enterobacteriales	GU968178
34	O1/7	*Eggerthella lenta*	96	Actinobacteria	Coriobacteridae	Coriobacteriales	GU968179
35	O1/7	*S. flexneri*	98	Proteobacteria	Gammaproteobacteria	Enterobacteriales	GU968180
39	O2/6	*Clostridium scindens*	98	Firmicutes	Clostridia	Clostridiales	GU968181
42	O2/7	*Ruminococcus* sp.	96	Firmicutes	Clostridia	Clostridiales	One strand only
45	V1/5	*E. coli*	98	Proteobacteria	Gammaproteobacteria	Enterobacteriales	GU968182
46	V1/5	*E. coli*	98	Proteobacteria	Gammaproteobacteria	Enterobacteriales	GU968183
48	V1/5	*E. coli*	99	Proteobacteria	Gammaproteobacteria	Enterobacteriales	GU968184
49	V1/5	*E. coli*	99	Proteobacteria	Gammaproteobacteria	Enterobacteriales	GU968185
50	V1/6	*E. coli*	99	Proteobacteria	Gammaproteobacteria	Enterobacteriales	885 bp

## Discussion

The measurements made here of rates of NH_3_ production from different amino acid-containing substrates, the influence of monensin on these rates, and the properties of bacteria isolated on the basis of being able to grow on Trypticase have important implications for understanding the biochemistry and microbial ecology of amino acid metabolism, and therefore the production of potentially hazardous products that can be formed from amino acids and related nitrogenous compounds in the human colon [2]. These results add to the substantial body of knowledge generated by Smith and Macfarlane [1,8-11,20] in the following respects. Ammonia production from peptides and amino acids was compared in diluted fresh samples of faeces in a similar way, with very similar results to earlier studies. However, utilization of individual amino acids from peptides was also compared, using faecal samples from both vegetarians and omnivorous donors. The differences may be explained by different permease mechanisms for peptides and amino acids. The effects of monensin on NH_3_ production and amino acid dissimilation were shown, providing clues about the biochemistry and microbial ecology of amino acid dissimilation. Finally, the bacteria that were enriched by growth on peptides or amino acids as energy source were isolated and identified based on 16S rRNA gene sequences. Similar methodology in the rumen revealed the HAP population, with significant implications for animal nutrition. The results imply that, unlike in the rumen, there is no significant population of ‘hyper-ammonia-producing’ bacteria [18]. Instead, the species that were enriched by growth on peptides and amino acids in the absence of carbohydrates include several pathogenic species that have important implications for health.

Ammonia production rates from Trypticase were higher than from casein or from a corresponding amino acid mixture. Casein is a protein that is atypically susceptible to proteolytic degradation, so it may be concluded that the first step of protein degradation to NH_3_ in the intestine, namely proteolysis, would generally be rate-limiting. Smith & Macfarlane [1] also noted that NH_3_ production was greater from peptides than amino acids, and suggested that amino acid transport in the form of peptides would be more energy-efficient than free amino acids. NH_3_ production from amino acids was more sensitive to the ionophore, monensin, than from peptides. The greater sensitivity to monensin of amino acid compared to peptide metabolism presumably reflects differences in transport mechanisms into bacteria. Transport of peptides in bacteria occurs predominantly by the ABC superfamily of transporters, which use ATP to drive uptake [21,22], while amino acid transport is more commonly linked to proton or Na^+^ gradients [23]. As monensin catalyzes Na^+^/H^+^ antiport in susceptible bacteria [24,25], this ionophore would therefore affect ion-linked amino acid transport more than ATP-linked peptide transport.

Smith & Macfarlane [20] investigated the metabolism of individual amino acids and a few pairs of amino acids in slurries of human faecal bacteria, and found that Ser was much more rapidly degraded than other amino acids. The same authors investigated breakdown of a complete mixture of free amino acids added to a fermenter that had been inoculated with a suspension of human faecal bacteria. Ser was again degraded most rapidly, with Asp close behind, followed by Arg. Glu was lost at less than one-quarter of the rate of Asp. Aromatic amino acids were degraded most slowly. The results of the present study were fairly similar, with the major exceptions of Glu, which was broken down most rapidly of all amino acids, and Lys, which was third or fourth most rapidly degraded amino acid in our studies but among the very lowest in Smith & Macfarlane [1]. While there were differences between methods in the studies, none offers an obvious explanation for these differences. Also, it is not clear whether the routes of metabolism of relatively low concentrations of amino acids in a complete mixture and metabolized by a mixed microbiota would be the same as pure cultures metabolizing the amino acid as a single substrate. This may be particularly relevant to Glu, which can be metabolized either via the methylaspartate pathway in clostridia or the hydroxyglutamate pathway in other species [26,27], yet, in mixtures of amino acids in a mixed culture with lower concentrations of Glu, Glu is most probably deaminated or transaminated to α-oxoglutarate, which then enters and disperses into central metabolic pathways.

The pattern of utilization of different amino acids was similar whether the amino acids were free or added as peptides. This provides a major contrast to the rumen, where peptide-bound amino acids are metabolized at different rates to free amino acids and in a different order [28,29]. Peptide uptake can predominate over amino acids in the ruminal bacteria of many animals, depending on the predominant microbiota that is present [30,31]. The present results with the human microbiota suggest that, at least in the individuals who provided samples here, amino acid utilizing bacteria are more dominant than peptide utilizers. The results with faecal samples from omnivores compared to vegetarians were inconclusive in terms of NH_3_ production, but the ranking order of dissimilation of different amino acids was similar.

The influence of monensin was different with different amino acids. Pro, Ala and Glu were inhibited most, with Asp and Lys affected only to a minor extent. Once again, the reason for this difference is unclear, but presumably reflects the inhibition of some transport systems and not others, or possibly a differential inhibition of species that metabolize different amino acids [17,18].

One of the principal aims of this work was to investigate if, by analogy with the rumen, HAP bacteria were present in the human colon. Conditions of low-carbohydrate, high-protein nutrient availability would favour bacteria able to derive energy from amino acids, particularly in the distal colon, but the general procedure of routinely adding sugars to growth media may have concealed these bacteria in culture-based studies. There had been a long-held assumption for the rumen that a large group of bacteria identified many years ago [32] was responsible for ruminal amino acid deamination. Russell and his colleagues at Cornell University challenged this assumption, and isolated less numerous, but much more active, asaccharolytic, obligately peptide-fermenting bacteria, the HAP species [18]. Growth of HAP bacteria was inhibited by monensin, while the more numerous NH_3_ producers were unaffected, yet NH_3_ production by the mixed ruminal microbiota was monensin-sensitive. The present paper suggests that the human microbiota has an NH_3_-producing activity about one-third that of the rumen [17]. Nevertheless, it is clear that a substantial fraction of NH_3_ production from peptides and amino acids is monensin-sensitive, so the possibility existed that HAP species were present in human colonic digesta.

Bacteria capable of growth on peptides as energy source were variable in number, averaging 3.5% of the total viable count. This proportion is somewhat higher than was found in ruminal digesta [16-18]. Actual numbers varied from 0.8 × 10^7^ to 3.5 × 10^8^ (g wet wt)^-1^, which compares with 10^11^ per g dry weight on peptone medium measured by Smith & Macfarlane [1]. Numbers capable of growth on amino acids were almost as high as those growing on Trypticase, which is a complete contrast to the rumen, where numbers of amino acids utilizers were two orders of magnitude less than Trypticase utilizers [17]. Thus, the bacterial population capable of using protein breakdown products in the human colon was more numerous than in the rumen, but less active, and differed in its much lower preference for peptides. As with the rumen, >90% of Trypticase utilizers were sensitive to inhibition by monensin, indicating that the bacteria were predominantly Gram-positive [33].

Smith & Macfarlane [1] enumerated amino acid-fermenting colonic bacteria in medium containing peptone, but did not isolate the bacteria concerned, concentrating on bacteria growing on individual or Stickland pairs of amino acids. The latter included mainly *Clostridium* species, with *Peptostreptococcus*, *Fusobacterium*, *Actinomyces*, *Bacteroides*, *Megasphaera* and *Propionibacterium* all represented. In the present study, the enrichments resulted in the isolation of several groups of bacteria. None was a HAP species, as all fermented glucose. Thus, the microbial ecology of the rumen and the human colon are fundamentally different in this respect. The species were also different to those isolated by Smith & Macfarlane [1]. *Clostridium* and *Bacteroides* were similarly predominant, though the species were different. Notably, one of the bacteria enriched was *C*. *perfringens*, which is a pathogen in animal species and man [34,35]. One might conclude, therefore, that individuals consuming a low-carbohydrate, high-protein weight loss diet would be vulnerable to increased numbers of pathogens in the intestine, as well as the better characterized genotoxic and inflammatory products of amino acid catabolism [2].

## Conclusions

The metabolism of peptides and amino acids by human faecal bacteria has many parallels with similar metabolism in the rumen, except that the bacteria that grow on these substrates are not specialist asaccharolytic (HAP) species. Instead, they tend to be pathogens. Thus, the implication is that when protein is the main substrate for intestinal bacteria, not only are the products of protein fermentation toxic, the bacteria enriched by these conditions may be harmful.

## Methods

### Donors

These experiments were carried out in compliance with the Helsinki Declaration of Ethical Principles for Medical Research Involving Human Subjects (http://www.wma.net/en/30publications/10policies/b3/index.html). Ethical approval was granted by the North of Scotland Research Ethics Committee and all subjects provided informed signed consent. Fresh faeces were obtained from three omnivorous (O1 – O3) and three vegetarian (V1 – V3) donors. No specific diets were given. O1 and O2 were 33-year-old females, O3 was a 47-year-old female, V1 and V3 were 56- and 26-year-old males, and V2 was a 43-year-old female. None had received antibiotic therapy for 3 months before samples were given. All samples were used fresh.

### Measurement of ammonia production in faecal suspensions *in vitro*

Faecal samples were diluted 1:10 wet weight in Chen & Russell basal medium [36], the suspension was homogenized in a stomacher, and 10 ml were added, under CO_2_, to Hungate-type tubes containing 200 mg substrate with or without 10 μl ethanol or 10 μl 5 mM monensin (Sigma, Poole, Dorset, UK) in ethanol. This monensin concentration (5 μM) was based on that used by Chen & Russell [16,37], which is similar to the estimated monensin concentration *in vivo*[38]. Tubes were incubated *in vitro* under CO_2_ in a water bath at 37°C. Substrates included casein (Sigma), Trypticase® peptone (Becton Dickinson Microbiology Systems, Cockeysville, MD 21030), and an amino acids mixture based on the composition of casein. The amino acids mixture comprised Gibco casein hydrolysate No. 5 (Life Technologies Ltd, Paisley, UK) plus added L-tryptophan (0.87%), L-methionine (0.17%) and L-cysteine (0.14%). One-ml samples were removed at 0, 2, 4, 6 and 8 h into 1.5-ml microcentrifuge tubes containing 0.25 ml 25% TCA. Samples were stored at 4°C, then centrifuged at 27,000 *g* for 20 min and ammonia was measured on supernatants. Ammonia was determined in the supernatant fluid by an automated phenol-hypochlorite method [39] and protein was determined on the acid precipitate using the Folin reagent [40]. For amino acids analysis, aliquots from the supernatant were dried under vacuum and hydrolysed by a vapour phase method (constant boiling HCl, 110°C, 18 h) and then derivatized with phenylisothionate and analysed by HPLC [41].

### Bacterial counts

Samples of faecal suspensions were diluted serially ten-fold under CO_2_ in a vitamins/minerals medium with no carbohydrate source, based on that described by Chen & Russell [36]. The basal medium contained, per liter, 292 mg of K_2_HPO_4_, 292 mg of KH_2_PO_4_, 480 mg of Na_2_SO_4_, 480 mg of NaCl, 100 mg of MgSO_4_.7H_2_O, 55 mg anhydrous CaCl_2_, 1.0 ml of 0.1% resazurin, 600 mg of cysteine hydrochloride and vitamins and minerals solutions [36]. The medium was adjusted to pH 7.0 before autoclaving. These dilutions were used to inoculate (1%, v/v) Hungate tubes containing four different liquid media: A, complete liquid form of medium M2 [42]; B, basal + 15 g/liter Trypticase® peptone (Becton Dickinson Microbiology Systems, Cockeysville, MD 21030); C, medium B + 5 μM monensin; D, basal + 15 g l^-1^ Casamino acids (Difco, Becton Dickinson Europe, 38241 Meylan cedex, France). Five tubes were inoculated for each dilution, the gas phase was 100% CO_2_, and tubes were incubated at 37°C. The optical density at 650 nm was determined periodically using an LKB Novaspec spectrophotometer. Numbers were calculated using most-probable-number tables [43], using a threshold of 0.1 as positive for growth.

### Isolation and identification of peptide and amino acid utilisers

Cultures from the highest dilutions in medium B and D were passaged once more in the same medium as before, then streaked on the corresponding agar medium. Individual colonies of different morphology were picked off, transferred to the same medium and incubated at 37°C. The isolation was then repeated. The ability of isolates to use glucose for growth was examined by inoculating the isolates into medium B or D to which 0.1% glucose had been added, and comparing the optical density after 48 h incubation with the corresponding optical density in unmodified medium. DNA was extracted from bacteria grown in both media using QIAmp DNA stool mini kit (Qiagen, Crawley, West Sussex, UK). Sequences of 16S rRNA genes were amplified using universal primers, fD1 and rP2 [44], in a mixture that contained 0.6 μM of each of the primers, 100 μM of each of the dNTPs, 2.5 mM MgCl_2_ in 1× buffer and 0.025 U/ml Taq polymerase (Bioline Ltd, London, UK). Amplification was carried out using a BioRad Icycler and the following programme: 94°C for 10 min; 35 cycles of 94°C for 1 min, 60°C for 1 min, 72°C for 2 min; then 72°C for 10 min, then 4°C. Amplification was confirmed by agarose gel electrophoresis. PCR products were cleaned up using Wizard^R^ SV Gel & PCR Clean-up system (Promega). Sequencing was carried out with fD1 and rP2 primers as before, with 2 further forward (926f, 519f) and 2 reverse primers (926r, 519r) based on Lane *et al*. [45]. Sequences were assembled with the Lasergene programme [46] and bacteria identified with NCBI Blastn. Where samples did not produce long enough sequences, amplified DNA was cloned into the PCR®2.1-TOPO vector (Invitrogen BV, Leek, the Netherlands). Plasmids were isolated from recombinant colonies using Wizard®*Plus* SV Miniprep DNA Purification System (Promega). Plasmids were checked for inserts by amplification with M13F and M13R primers followed by agarose gel electrophoresis. Plasmids which contained inserts were sequenced using M13F and M13R primers initially then all 6 primers as used before. Sequences were assembled and identified as before. Full length or near full length 16S rRNA genes sequences have been deposited in the GenBank database, with accession numbers GU968162-GU968185.

### Data analysis

Ammonia production rates were analysed by hierarchical Analysis of Variance, with a between and within subject stratum, with factors for diet (omnivore *vs* vegetarian), medium (Trypticase vs amino acids) and monensin and their interactions.

Production was linear during the incubations and rates of NH_3_ production were determined by linear regression and compared by ANOVA in Microsoft Excel.

## Abbreviations

HAP: ‘Hyper-ammonia-producing’ bacteria.

## Competing interests

The authors declare that they have no competing interests.

## Authors' contributions

AJR carried out most of the experimental work, organised the volunteers and suggested corrections to the manuscript. NMcK carried out some experimental work, advised on techniques and suggested modifications to the manuscript. RJW initiated the work, designed the experiments and wrote the manuscript. All authors read and approved the final manuscript.
